# Adverse Childhood Events Are Associated With Physical, Psychological, and Cognitive Health in Menopause

**DOI:** 10.1093/geroni/igaf122.1805

**Published:** 2025-12-31

**Authors:** Emma Muller, Pilar Thangwaritorn, Jaime Perales-Puchalt, Melanie Meister, Amber Watts

**Affiliations:** University of Kansas, Lawrence, Kansas, United States; University of Kansas, Lawrence, Kansas, United States; University of Kansas Medical Center, Kansas City, Kansas, United States; University of Kansas School of Medicine, Kansas City, Kansas, United States; University of Kansas, Lawrence, Kansas, United States

## Abstract

Adverse childhood experiences (ACEs) can have long term impacts on women’s physical and psychological health and cognition. Despite growing evidence linking ACEs to negative health outcomes, the specific effects on women in midlife are underexplored. We present findings from an online survey of women (N = 98) hypothesizing that higher ACES would be associated with more symptoms reported during the menopause transition. Measures include the ACE-Q, PROMIS depression and anxiety scales, self-reported cognitive complaints, and Pittsburgh Sleep Quality Index (PSQI). Our approach uniquely compares methods of characterizing childhood adversity (number vs. type of exposures). Women from the purposive sample (53.1% non-Hispanic white, 24.5% Hispanic/Latina, 17.3% African American, 8.2% rural-dwelling, 63.3% college-educated and above) were between the ages of 40-62 years of age (M age = 49.47). Our cross-sectional analysis showed higher ACE scores were associated with increased odds of reporting poor memory (p = 0.012, OR = 1.347) and difficulty sleeping (p = 0.009, OR = 1.44) adjusting for covariates. ACE scores and Anxiety scores showed a weak, but statistically significant positive correlation (r = 0.226, p = 0.036) that was attenuated below the level of significance in multivariate models. Specific adverse experiences such as emotional neglect and abuse were significantly associated with menopause symptoms, including difficulty concentrating and crying spells, and other symptoms including bladder issues, pain, bloating, anxiety and depression in univariate models (r = 0.316–0.626, p < 0.05). Our findings align with lifespan theoretical perspectives suggesting early life events have lifelong cumulative effects. They also have implications for clinical practice in the menopause transition, including screening for psychological and cognitive symptoms.

